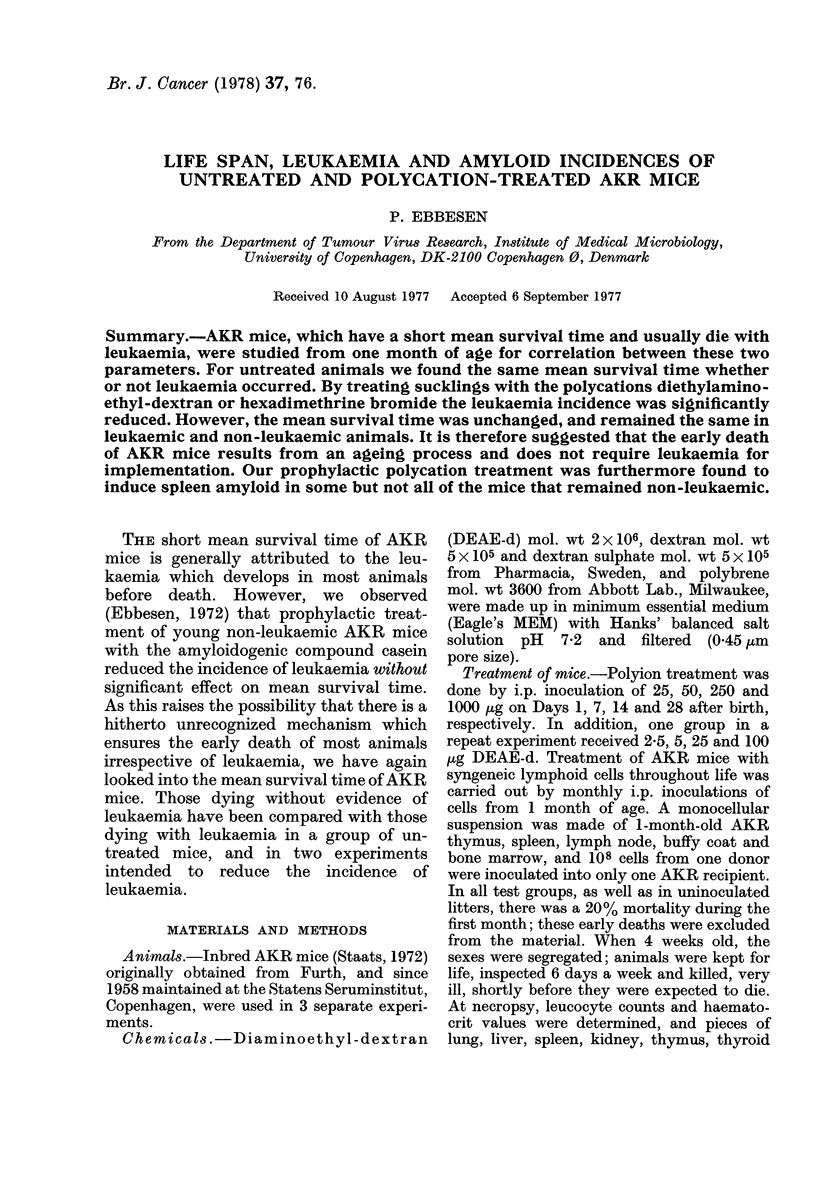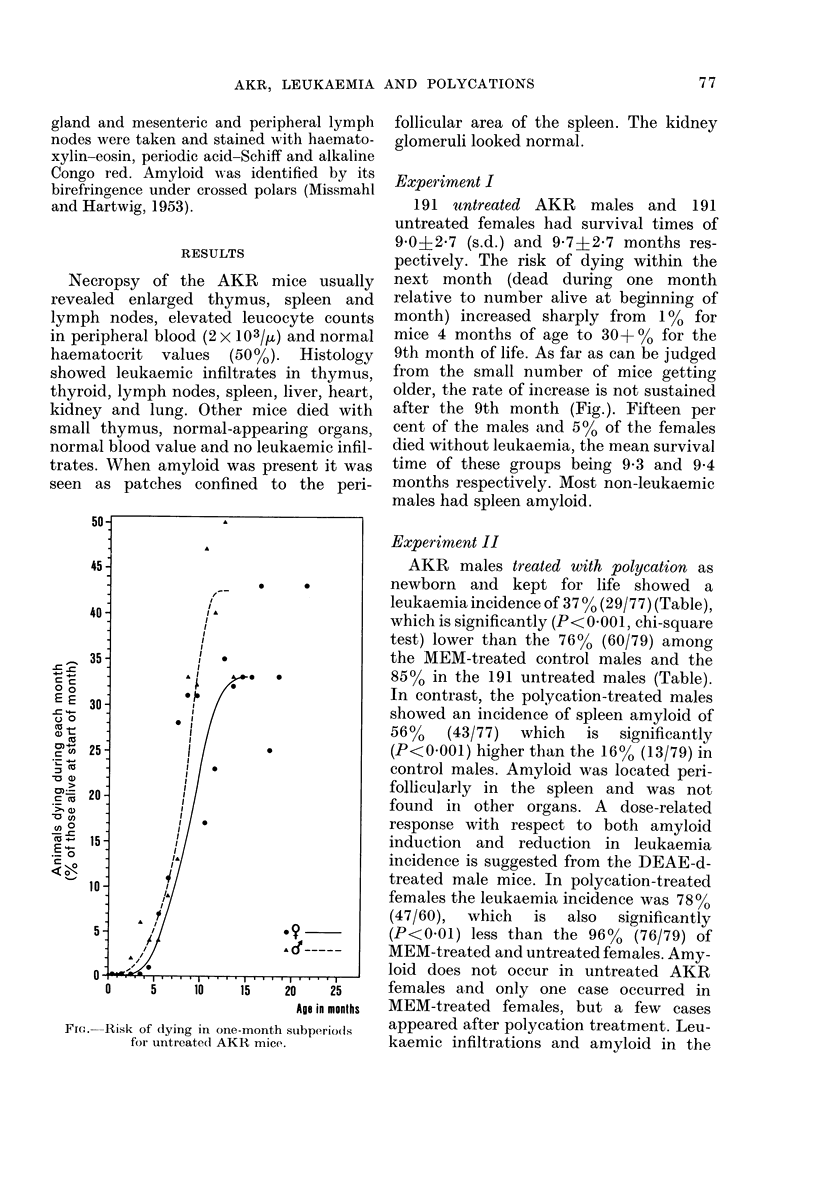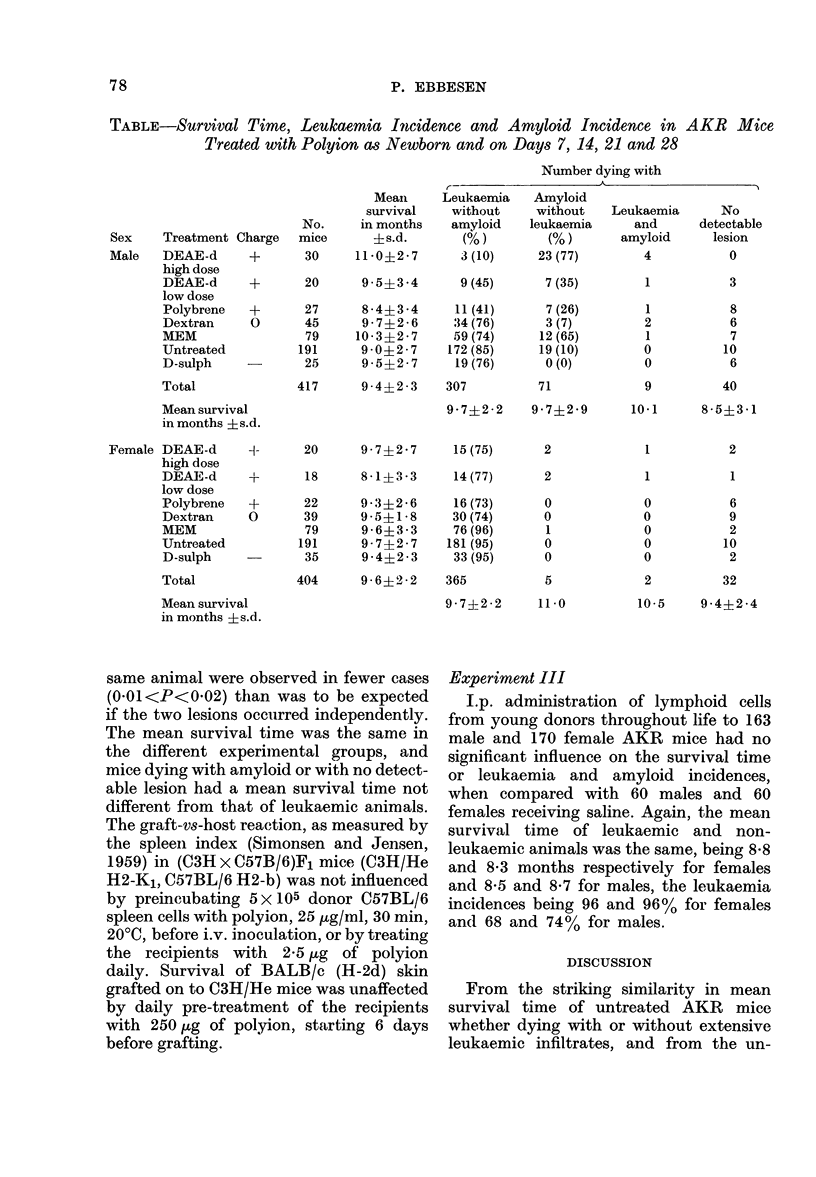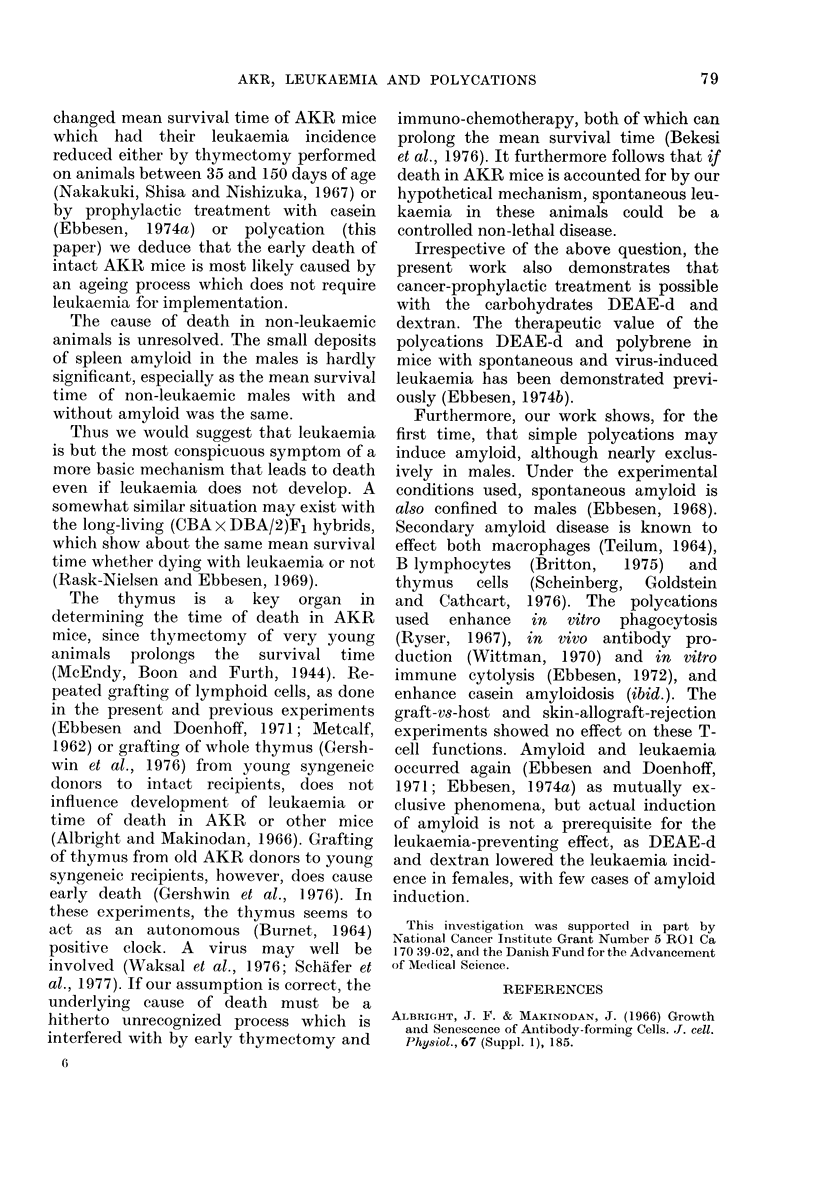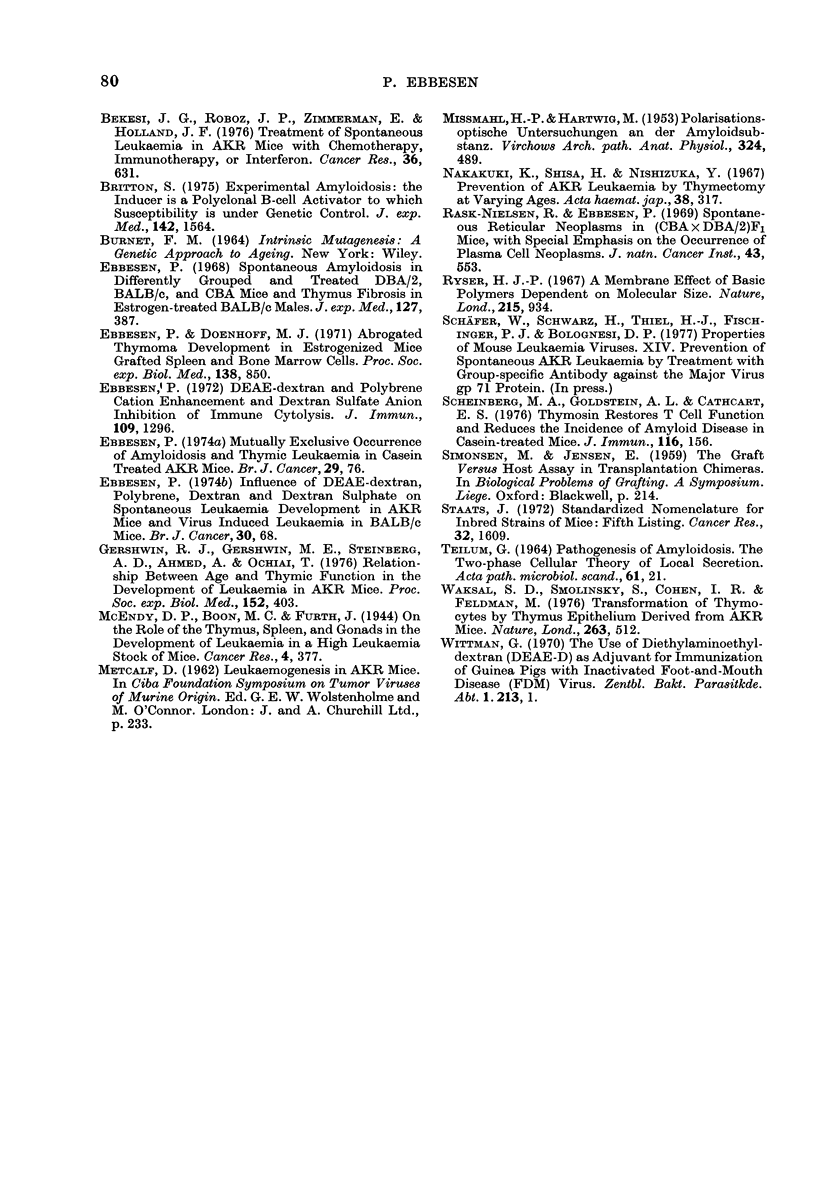# Life span, leukaemia and amyloid incidences of untreated and polycation-treated AKR mice.

**DOI:** 10.1038/bjc.1978.11

**Published:** 1978-01

**Authors:** P. Ebbesen

## Abstract

AKR mice, which have a short mean survival time and usually die with leukaemia, were studied from one month of age for correlation between these two parameters. For untreated animals we found the same mean survival time whether or not leukaemia occurred. By treating sucklings with the polycations diethylaminoethyl-dextran or hexadimethrine bromide the leukaemia incidence was significantly reduced. However, the mean survival time was unchanged, and remained the same in leukaemic and non-leukaemic animals. It is therefore suggested that the early death of AKR mice results from an ageing process and does not require leukaemia for implementation. Our prophylactic polycation treatment was furthermore found to induce spleen amyloid in some but not all of the mice that remained non-leukaemic.


*Br. J. Cancer* (1978) **37**, 76.

### LIFE SPAN, LEUKAEMIA AND AMYLOID INCIDENCES OF UNTREATED AND POLYCATION-TREATED AKR MICE

P. EBBESEN

*From the Department of Tumour Virus Research, Institute of Medical Microbiology, University of Copenhagen, DK-2100 Copenhagen Ø, Denmark*

Received 10 August 1977 Accepted 6 September 1977

**Summary.—AKR mice, which have a short mean survival time and usually die with leukaemia, were studied from one month of age for correlation between these two parameters. For untreated animals we found the same mean survival time whether or not leukaemia occurred. By treating sucklings with the polycations diethylamino-ethyl-dextran or hexadimethrine bromide the leukaemia incidence was significantly reduced. However, the mean survival time was unchanged, and remained the same in leukaemic and non-leukaemic animals. It is therefore suggested that the early death of AKR mice results from an ageing process and does not require leukaemia for implementation. Our prophylactic polycation treatment was furthermore found to induce spleen amyloid in some but not all of the mice that remained non-leukaemic.**

THE short mean survival time of AKR mice is generally attributed to the leukaemia which develops in most animals before death. However, we observed (Ebbesen, 1972) that prophylactic treatment of young non-leukaemic AKR mice with the amyloidogenic compound casein reduced the incidence of leukaemia *without* significant effect on mean survival time. As this raises the possibility that there is a hitherto unrecognized mechanism which ensures the early death of most animals irrespective of leukaemia, we have again looked into the mean survival time of AKR mice. Those dying without evidence of leukaemia have been compared with those dying with leukaemia in a group of untreated mice, and in two experiments intended to reduce the incidence of leukaemia.

### MATERIALS AND METHODS

*Animals.*—Inbred AKR mice (Staats, 1972) originally obtained from Furth, and since 1958 maintained at the Statens Seruminstitut, Copenhagen, were used in 3 separate experiments.

*Chemicals.*—Diaminoethyl-dextran (DEAE-d) mol. wt $2\times10^6$, dextran mol. wt $5\times10^5$ and dextran sulphate mol. wt $5\times10^5$ from Pharmacia, Sweden, and polybrene mol. wt 3600 from Abbott Lab., Milwaukee, were made up in minimum essential medium (Eagle's MEM) with Hanks' balanced salt solution pH 7·2 and filtered (0·45 $\mu$m pore size).

*Treatment of mice.*—Polyion treatment was done by i.p. inoculation of 25, 50, 250 and 1000 $\mu$g on Days 1, 7, 14 and 28 after birth, respectively. In addition, one group in a repeat experiment received 2·5, 5, 25 and 100 $\mu$g DEAE-d. Treatment of AKR mice with syngeneic lymphoid cells throughout life was carried out by monthly i.p. inoculations of cells from 1 month of age. A monocellular suspension was made of 1-month-old AKR thymus, spleen, lymph node, buffy coat and bone marrow, and $10^8$ cells from one donor were inoculated into only one AKR recipient. In all test groups, as well as in uninoculated litters, there was a 20% mortality during the first month; these early deaths were excluded from the material. When 4 weeks old, the sexes were segregated; animals were kept for life, inspected 6 days a week and killed, very ill, shortly before they were expected to die. At necropsy, leucocyte counts and haematocrit values were determined, and pieces of lung, liver, spleen, kidney, thymus, thyroid gland and mesenteric and peripheral lymph nodes were taken and stained with haematoxylin–eosin, periodic acid–Schiff and alkaline Congo red. Amyloid was identified by its birefringence under crossed polars (Missmahl and Hartwig, 1953).

### RESULTS

Necropsy of the AKR mice usually revealed enlarged thymus, spleen and lymph nodes, elevated leucocyte counts in peripheral blood ($2 \times 10^3/\mu$) and normal haematocrit values (50%). Histology showed leukaemic infiltrates in thymus, thyroid, lymph nodes, spleen, liver, heart, kidney and lung. Other mice died with small thymus, normal-appearing organs, normal blood value and no leukaemic infiltrates. When amyloid was present it was seen as patches confined to the perifollicular area of the spleen. The kidney glomeruli looked normal.

FIG.—Risk of dying in one-month subperiods for untreated AKR mice.

*Experiment I*

191 *untreated* AKR males and 191 untreated females had survival times of $9{\cdot}0 \pm 2{\cdot}7$ (s.d.) and $9{\cdot}7 \pm 2{\cdot}7$ months respectively. The risk of dying within the next month (dead during one month relative to number alive at beginning of month) increased sharply from 1% for mice 4 months of age to 30+% for the 9th month of life. As far as can be judged from the small number of mice getting older, the rate of increase is not sustained after the 9th month (Fig.). Fifteen per cent of the males and 5% of the females died without leukaemia, the mean survival time of these groups being 9·3 and 9·4 months respectively. Most non-leukaemic males had spleen amyloid.

*Experiment II*

AKR males *treated with polycation* as newborn and kept for life showed a leukaemia incidence of 37% (29/77) (Table), which is significantly ($P < 0{\cdot}001$, chi-square test) lower than the 76% (60/79) among the MEM-treated control males and the 85% in the 191 untreated males (Table). In contrast, the polycation-treated males showed an incidence of spleen amyloid of 56% (43/77) which is significantly ($P < 0{\cdot}001$) higher than the 16% (13/79) in control males. Amyloid was located perifollicularly in the spleen and was not found in other organs. A dose-related response with respect to both amyloid induction and reduction in leukaemia incidence is suggested from the DEAE-d-treated male mice. In polycation-treated females the leukaemia incidence was 78% (47/60), which is also significantly ($P < 0{\cdot}01$) less than the 96% (76/79) of MEM-treated and untreated females. Amyloid does not occur in untreated AKR females and only one case occurred in MEM-treated females, but a few cases appeared after polycation treatment. Leukaemic infiltrations and amyloid in the

TABLE—*Survival Time, Leukaemia Incidence and Amyloid Incidence in AKR Mice Treated with Polyion as Newborn and on Days 7, 14, 21 and 28*

| Sex | Treatment | Charge | No. mice | Mean survival in months ±s.d. | Number dying with: Leukaemia without amyloid (%) | Amyloid without leukaemia (%) | Leukaemia and amyloid | No detectable lesion |
|---|---|---|---|---|---|---|---|---|
| Male | DEAE-d high dose | + | 30 | 11·0±2·7 | 3 (10) | 23 (77) | 4 | 0 |
| | DEAE-d low dose | + | 20 | 9·5±3·4 | 9 (45) | 7 (35) | 1 | 3 |
| | Polybrene | + | 27 | 8·4±3·4 | 11 (41) | 7 (26) | 1 | 8 |
| | Dextran | O | 45 | 9·7±2·6 | 34 (76) | 3 (7) | 2 | 6 |
| | MEM | | 79 | 10·3±2·7 | 59 (74) | 12 (65) | 1 | 7 |
| | Untreated | | 191 | 9·0±2·7 | 172 (85) | 19 (10) | 0 | 10 |
| | D-sulph | — | 25 | 9·5±2·7 | 19 (76) | 0 (0) | 0 | 6 |
| | Total | | 417 | 9·4±2·3 | 307 | 71 | 9 | 40 |
| | Mean survival in months ±s.d. | | | | 9·7±2·2 | 9·7±2·9 | 10·1 | 8·5±3·1 |
| Female | DEAE-d high dose | + | 20 | 9·7±2·7 | 15 (75) | 2 | 1 | 2 |
| | DEAE-d low dose | + | 18 | 8·1±3·3 | 14 (77) | 2 | 1 | 1 |
| | Polybrene | + | 22 | 9·3±2·6 | 16 (73) | 0 | 0 | 6 |
| | Dextran | O | 39 | 9·5±1·8 | 30 (74) | 0 | 0 | 9 |
| | MEM | | 79 | 9·6±3·3 | 76 (96) | 1 | 0 | 2 |
| | Untreated | | 191 | 9·7±2·7 | 181 (95) | 0 | 0 | 10 |
| | D-sulph | — | 35 | 9·4±2·3 | 33 (95) | 0 | 0 | 2 |
| | Total | | 404 | 9·6±2·2 | 365 | 5 | 2 | 32 |
| | Mean survival in months ±s.d. | | | | 9·7±2·2 | 11·0 | 10·5 | 9·4±2·4 |

same animal were observed in fewer cases ($0{\cdot}01<P<0{\cdot}02$) than was to be expected if the two lesions occurred independently. The mean survival time was the same in the different experimental groups, and mice dying with amyloid or with no detectable lesion had a mean survival time not different from that of leukaemic animals. The graft-*vs*-host reaction, as measured by the spleen index (Simonsen and Jensen, 1959) in (C3H×C57B/6)$F_1$ mice (C3H/He H2-$K_1$, C57BL/6 H2-b) was not influenced by preincubating $5\times10^5$ donor C57BL/6 spleen cells with polyion, 25 μg/ml, 30 min, 20°C, before i.v. inoculation, or by treating the recipients with 2·5 μg of polyion daily. Survival of BALB/c (H-2d) skin grafted on to C3H/He mice was unaffected by daily pre-treatment of the recipients with 250 μg of polyion, starting 6 days before grafting.

*Experiment III*

I.p. administration of lymphoid cells from young donors throughout life to 163 male and 170 female AKR mice had no significant influence on the survival time or leukaemia and amyloid incidences, when compared with 60 males and 60 females receiving saline. Again, the mean survival time of leukaemic and non-leukaemic animals was the same, being 8·8 and 8·3 months respectively for females and 8·5 and 8·7 for males, the leukaemia incidences being 96 and 96% for females and 68 and 74% for males.

### DISCUSSION

From the striking similarity in mean survival time of untreated AKR mice whether dying with or without extensive leukaemic infiltrates, and from the unchanged mean survival time of AKR mice which had their leukaemia incidence reduced either by thymectomy performed on animals between 35 and 150 days of age (Nakakuki, Shisa and Nishizuka, 1967) or by prophylactic treatment with casein (Ebbesen, 1974*a*) or polycation (this paper) we deduce that the early death of intact AKR mice is most likely caused by an ageing process which does not require leukaemia for implementation.

The cause of death in non-leukaemic animals is unresolved. The small deposits of spleen amyloid in the males is hardly significant, especially as the mean survival time of non-leukaemic males with and without amyloid was the same.

Thus we would suggest that leukaemia is but the most conspicuous symptom of a more basic mechanism that leads to death even if leukaemia does not develop. A somewhat similar situation may exist with the long-living (CBA × DBA/2)$F_1$ hybrids, which show about the same mean survival time whether dying with leukaemia or not (Rask-Nielsen and Ebbesen, 1969).

The thymus is a key organ in determining the time of death in AKR mice, since thymectomy of very young animals prolongs the survival time (McEndy, Boon and Furth, 1944). Repeated grafting of lymphoid cells, as done in the present and previous experiments (Ebbesen and Doenhoff, 1971; Metcalf, 1962) or grafting of whole thymus (Gershwin *et al.*, 1976) from young syngeneic donors to intact recipients, does not influence development of leukaemia or time of death in AKR or other mice (Albright and Makinodan, 1966). Grafting of thymus from old AKR donors to young syngeneic recipients, however, does cause early death (Gershwin *et al.*, 1976). In these experiments, the thymus seems to act as an autonomous (Burnet, 1964) positive clock. A virus may well be involved (Waksal *et al.*, 1976; Schäfer *et al.*, 1977). If our assumption is correct, the underlying cause of death must be a hitherto unrecognized process which is interfered with by early thymectomy and immuno-chemotherapy, both of which can prolong the mean survival time (Bekesi *et al.*, 1976). It furthermore follows that *if* death in AKR mice is accounted for by our hypothetical mechanism, spontaneous leukaemia in these animals could be a controlled non-lethal disease.

Irrespective of the above question, the present work also demonstrates that cancer-prophylactic treatment is possible with the carbohydrates DEAE-d and dextran. The therapeutic value of the polycations DEAE-d and polybrene in mice with spontaneous and virus-induced leukaemia has been demonstrated previously (Ebbesen, 1974*b*).

Furthermore, our work shows, for the first time, that simple polycations may induce amyloid, although nearly exclusively in males. Under the experimental conditions used, spontaneous amyloid is *also* confined to males (Ebbesen, 1968). Secondary amyloid disease is known to effect both macrophages (Teilum, 1964), B lymphocytes (Britton, 1975) and thymus cells (Scheinberg, Goldstein and Cathcart, 1976). The polycations used enhance *in vitro* phagocytosis (Ryser, 1967), *in vivo* antibody production (Wittman, 1970) and *in vitro* immune cytolysis (Ebbesen, 1972), and enhance casein amyloidosis (*ibid.*). The graft-*vs*-host and skin-allograft-rejection experiments showed no effect on these T-cell functions. Amyloid and leukaemia occurred again (Ebbesen and Doenhoff, 1971; Ebbesen, 1974*a*) as mutually exclusive phenomena, but actual induction of amyloid is not a prerequisite for the leukaemia-preventing effect, as DEAE-d and dextran lowered the leukaemia incidence in females, with few cases of amyloid induction.

This investigation was supported in part by National Cancer Institute Grant Number 5 RO1 Ca 170 39-02, and the Danish Fund for the Advancement of Medical Science.

### REFERENCES

Albright, J. F. & Makinodan, J. (1966) Growth and Senescence of Antibody-forming Cells. *J. cell. Physiol.*, **67** (Suppl. 1), 185.

BEKESI, J. G., ROBOZ, J. P., ZIMMERMAN, E. & HOLLAND, J. F. (1976) Treatment of Spontaneous Leukaemia in AKR Mice with Chemotherapy, Immunotherapy, or Interferon. *Cancer Res.*, **36**, 631.

BRITTON, S. (1975) Experimental Amyloidosis: the Inducer is a Polyclonal B-cell Activator to which Susceptibility is under Genetic Control. *J. exp. Med.*, **142**, 1564.

BURNET, F. M. (1964) *Intrinsic Mutagenesis: A Genetic Approach to Ageing.* New York: Wiley.

EBBESEN, P. (1968) Spontaneous Amyloidosis in Differently Grouped and Treated DBA/2, BALB/c, and CBA Mice and Thymus Fibrosis in Estrogen-treated BALB/c Males. *J. exp. Med.*, **127**, 387.

EBBESEN, P. & DOENHOFF, M. J. (1971) Abrogated Thymoma Development in Estrogenized Mice Grafted Spleen and Bone Marrow Cells. *Proc. Soc. exp. Biol. Med.*, **138**, 850.

EBBESEN,' P. (1972) DEAE-dextran and Polybrene Cation Enhancement and Dextran Sulfate Anion Inhibition of Immune Cytolysis. *J. Immun.*, **109**, 1296.

EBBESEN, P. (1974*a*) Mutually Exclusive Occurrence of Amyloidosis and Thymic Leukaemia in Casein Treated AKR Mice. *Br. J. Cancer*, **29**, 76.

EBBESEN, P. (1974*b*) Influence of DEAE-dextran, Polybrene, Dextran and Dextran Sulphate on Spontaneous Leukaemia Development in AKR Mice and Virus Induced Leukaemia in BALB/c Mice. *Br. J. Cancer*, **30**, 68.

GERSHWIN, R. J., GERSHWIN, M. E., STEINBERG, A. D., AHMED, A. & OCHIAI, T. (1976) Relationship Between Age and Thymic Function in the Development of Leukaemia in AKR Mice. *Proc. Soc. exp. Biol. Med.*, **152**, 403.

MCENDY, D. P., BOON, M. C. & FURTH, J. (1944) On the Role of the Thymus, Spleen, and Gonads in the Development of Leukaemia in a High Leukaemia Stock of Mice. *Cancer Res.*, **4**, 377.

METCALF, D. (1962) Leukaemogenesis in AKR Mice. In *Ciba Foundation Symposium on Tumor Viruses of Murine Origin.* Ed. G. E. W. Wolstenholme and M. O'Connor. London: J. and A. Churchill Ltd., p. 233.

MISSMAHL, H.-P. & HARTWIG, M. (1953) Polarisationsoptische Untersuchungen an der Amyloidsubstanz. *Virchows Arch. path. Anat. Physiol.*, **324**, 489.

NAKAKUKI, K., SHISA, H. & NISHIZUKA, Y. (1967) Prevention of AKR Leukaemia by Thymectomy at Varying Ages. *Acta haemat. jap.*, **38**, 317.

RASK-NIELSEN, R. & EBBESEN, P. (1969) Spontaneous Reticular Neoplasms in (CBA$\times$DBA/2)$F_1$ Mice, with Special Emphasis on the Occurrence of Plasma Cell Neoplasms. *J. natn. Cancer Inst.*, **43**, 553.

RYSER, H. J.-P. (1967) A Membrane Effect of Basic Polymers Dependent on Molecular Size. *Nature, Lond.*, **215**, 934.

SCHÄFER, W., SCHWARZ, H., THIEL, H.-J., FISCHINGER, P. J. & BOLOGNESI, D. P. (1977) Properties of Mouse Leukaemia Viruses. XIV. Prevention of Spontaneous AKR Leukaemia by Treatment with Group-specific Antibody against the Major Virus gp 71 Protein. (In press.)

SCHEINBERG, M. A., GOLDSTEIN, A. L. & CATHCART, E. S. (1976) Thymosin Restores T Cell Function and Reduces the Incidence of Amyloid Disease in Casein-treated Mice. *J. Immun.*, **116**, 156.

SIMONSEN, M. & JENSEN, E. (1959) The Graft *Versus* Host Assay in Transplantation Chimeras. In *Biological Problems of Grafting. A Symposium. Liege.* Oxford: Blackwell, p. 214.

STAATS, J. (1972) Standardized Nomenclature for Inbred Strains of Mice: Fifth Listing. *Cancer Res.*, **32**, 1609.

TEILUM, G. (1964) Pathogenesis of Amyloidosis. The Two-phase Cellular Theory of Local Secretion. *Acta path. microbiol. scand.*, **61**, 21.

WAKSAL, S. D., SMOLINSKY, S., COHEN, I. R. & FELDMAN, M. (1976) Transformation of Thymocytes by Thymus Epithelium Derived from AKR Mice. *Nature, Lond.*, **263**, 512.

WITTMAN, G. (1970) The Use of Diethylaminoethyl-dextran (DEAE-D) as Adjuvant for Immunization of Guinea Pigs with Inactivated Foot-and-Mouth Disease (FDM) Virus. *Zentbl. Bakt. Parasitkde. Abt.* **1.213**, 1.